# Prediction of the severity of child abuse using nationwide survey data from Child Guidance Centers in Japan: focus on infancy and preschool age

**DOI:** 10.3389/frcha.2024.1305811

**Published:** 2024-05-09

**Authors:** Yasukazu Ogai, Ryoko Nakajima-Yamaguchi, Hirotsuna Ohashi, Kentaro Niwa, Toyoo Sakurayama, Nobuaki Morita

**Affiliations:** ^1^ Social Psychiatry and Mental Health, Faculty of Medicine, University of Tsukuba, Ibaraki, Japan; ^2^ Ibaraki Children’s Hospital Children’s Medical and Cancer Research Center, Ibaraki, Japan; ^3^ Department of General Medicine, Japan Community Health Care Organization, Sendai Hospital, Miyagi, Japan; ^4^ Department of Child Development, School of Education, Sugiyama Jogakuen University, Aichi, Japan; ^5^ Former National Child Guidance Center Direction’s Association, Tokyo, Japan

**Keywords:** child abuse, child maltreatment, risk prediction, big-data analysis, severity

## Abstract

**Introduction:**

The present study conducted a secondary data analysis of a comprehensive survey from Child Guidance Centers in Japan to identify factors that are associated with child abuse severity in infancy (0–3 years, 1,868 cases) and preschool age (4–6 years, 1,529 cases). A predictive model for abuse severity was developed.

**Methods:**

The data originated from a nationwide survey that was conducted in April 2013, consisting of details of abuse cases, including child characteristics, abuser attributes, and family situation. Abuse severity was assessed on a five-level scale (suspected, mild, moderate, severe, and life-threatening) that was converted into a binary outcome. Logistic regression analysis was used to create a predictive model using two-thirds of the data, which was validated with the remaining third of the data.

**Results and discussion:**

As a result, in infancy, risks of severity increased with younger age of the abused child, physical abuse, neglect, witnessing domestic violence, and the involvement of Child Guidance Centers or hospitals in detection. The abuser's mental problems and cumulative child damage contributed to severity. For preschool age, similar factors applied, with additional risks that included abuse overlap and guardian separation. Cumulative abuser issues and child physical damage impacted severity. Validation yielded moderate prediction accuracy (areas under the curve: 0.703 and 0.714).

## 1 Introduction

Child abuse in Japan has become a significant issue in recent years. The number of cases that are reported to Child Guidance Centers has been on the rise, reaching 207,659 cases in FY2021, a nine-fold increase compared with the two prior decades (2001) (1). With this increase in cases, the workload of Child Guidance Center staff has become a significant concern. After the Child Welfare Law was revised in 2004, both Child Guidance Centers and local government officials must share some responsibility for child abuse consultation (2), but this is still insufficient to reduce the overall workload of Child Guidance Center staff (3). As a result, official responses and assistance may not adequately reach those in need, including potential at-risk groups for abuse (4).

The utilization of Big Data is being explored as a potential means to alleviate the workload of Child Guidance Center staff. In Japan, the Research Group of the Ministry of Health, Labor, and Welfare developed a guide for responding to child abuse (5) and a risk assessment tool (6) to address cases of child abuse, but there has been limited verification utilizing data and responses to the problem. Additionally, a comprehensive survey of cases that are reported by Child Guidance Centers nationwide is conducted every 5 years [e.g., (7)], but it is limited to only aggregate reports. For example, if the severity and temporary protection risks of abuse can be clarified using a large-scale nationwide database based on notification records of Child Guidance Centers, then this would provide professionals who are pressed to respond to cases within a short period of time with more information to make more effective decisions.

In the United States, the Differential Response Model (i.e., a response system based on initial risk assessment) was proposed. Depending on risk factors that are identified at the initial stage of notification, the response agency is assigned to either a child protection agency that focuses on an intervention response or a municipal child welfare facility that focuses on a support response (8, 9). Efforts have been made to use large-scale databases to make this decision and construct predictive risk models through multivariate analysis and machine learning (10), the aim of which is to achieve more efficient and highly accurate decision making for triage for which call centers are responsible.

In recent years, there has been a gradual increase in studies in Japan that aim to predict the risk of child maltreatment using large-scale data. For example, one study conducted a large-scale survey of mothers and children who participated in a 4-month health checkup by a local government and examined risk factors for physical abuse (11) and child shaking syndrome (12). Additionally, studies have examined risk factors for child shaking syndrome using a longitudinal survey of data that are reported as abuse to a Child Guidance Center in one municipality (13) and large datasets of cases that are reported as abuse to a child welfare consultation center in a different municipality using Bayesian networks to analyze characteristics of recurrent and non-recurrent cases (14). Artificial intelligence based child abuse case severity prediction software using data from local governments has also begun to be developed (15). The number of studies that utilize data from child response-related facilities nationwide is limited. Existing examples include a study (16) that analyzed risk factors for children who were temporarily placed in care and were then re-notified as abused using secondary data from a survey that was conducted among temporary shelters nationwide. Another study examined assessment items that predicted the risk of temporary custody using the Random Forest method of machine learning and making secondary use of data from a large-scale web survey that was conducted by the Ministry of Health, Labor, and Welfare that targeted Child Guidance Centers nationwide (17). Despite these attempts, however, the use of nationwide survey data in this field is still insufficient.

The Detailed Survey on Cases Notified to Child Guidance Centers as Child Abuse conducted by all Child Guidance Centers (7) is the only large-scale nationwide survey of its kind in Japan. This survey was conducted in 2013 and may not necessarily reflect the most recent situation. However, there are many results that are basically similar to those reported in a new national survey conducted in 2018 (18) in an almost identical format, and it can be said that the basic situation surrounding child abuse has not changed significantly. Also, the sample size is large compared to the most recent data, allowing for a variety of analyses to be conducted, including stratified analysis and model validation. Given the paucity of comprehensive studies utilizing national survey data in this area, our analysis is an important contribution to the field of child protection. At the same time, it asks a wide range of information needed to derive the severity of abuse (abuse severity and emergency responses such as temporary protection) and related risks. For example, in addition to basic information such as abuse type, the survey also asks about the characteristics of the abused child, the characteristics of the abuser, the situation of the abusive family household, and various types of damage resulting from the abuse. There is an accumulation of various previous studies on child abuse risk. In several systematic reviews that have been conducted (19, 20, 21), this nation-wide survey covers most of the items discussed as child abuse risk factors in the reviews.

This study will conduct a secondary analysis of this data to examine the risk factors that make child maltreatment cases more severe. This is not the risk of child abuse occurring, but rather the risk of whether the child abuse that does occur is severe enough to require immediate intervention. The identification of risk factors that increase child abuse severity will help child guidance center staff determine whether a case should be given priority, such as emergency intervention, during the initial response to child abuse. In this study, various peripheral factors after the occurrence of child abuse, such as the first discoverer of child abuse and physical damage caused by child abuse, are also included in the items examined in relation to abuse severity. This approach is supported by existing literature, which suggests that the source of abuse reports is associated with the severity of the case. For example, child abuse cases reported by medical institutions tend to involve more serious injuries (22), and cases in which physical injuries due to abuse are found at the time of notification signal serious physical abuse that requires immediate attention (23). Therefore, while these factors are associated with severe abuse, they also play a indicator in determining the severity of the situation, necessitating an emergency response by a professional. Incorporating a wide range of information available to the staff during the period between the occurrence of child abuse and when the staff is notified of the incident improves the assessment of situation. The outcome of this study will be child abuse severity (whether the severity of abuse is more severe than moderate abuse, which requires professional intervention). Since the factors associated with maltreatment severity are thought to vary with the age of the abused children, the data were analyzed by dividing them into five age groups.

The purpose of the present study was to conduct a secondary data analysis of the detailed survey of cases reported to the Child Guidance Center, and to examine variables that predict case severity ratings in infancy (0–3 years old) and preschool age (4–6 years old). We will also construct an exploratory model for predicting child abuse severity in each data set, and examine the validity of the model using a subset of the data.

## 2 Materials and methods

### 2.1 Database and outcome

A secondary data analysis was conducted of a nationwide survey of cases that were reported to Child Guidance Centers in Japan, with a sample size of 11,257 cases in April 2013, conducted by the National Association of Child Guidance Center Directors (7). This survey was conducted in 2013 with support from the Children's Future Foundation and in cooperation with Child Guidance Centers throughout Japan under Toyoo Sakurayama as the principal investigator. Utilization of the data and the secondary analysis were approved by the National Association of Child Guidance Center Directors and Medical Ethics Committee of University of Tsukuba.

In this survey, staff at Child Guidance Centers in response to notifications of suspected abuse recorded case information based on their records. As a result, 7,341 cases of abuse were confirmed and subsequently selected for analysis. The data were subsequently classified into five groups according to the age of the abused child, taking into account the possibility that the nature of abuse and risk factors that lead to abuse may vary by age of the abused child. The five age groups were infancy (0–3 years), preschool age (4–6 years), early school age (7–9 years), late school age (10–12 years), and adolescence (13–18 years). In this report, we examined the relationship between the severity of abuse and data for infancy and preschool age, respectively. There were 1,868 cases of abuse in infancy and 1,529 cases in preschool age.

The severity of abuse treated as an outcome in this study is assessed by Child Guidance Center staff who respond to notifications on a five-level scale (suspected abuse, mild abuse, moderate abuse, severe abuse, and life-threatening) based on the criteria that are set forth by the Ministry of Health, Labor, and Welfare (5). It is particularly important at the notification stage to determine whether or not a visit or other focused response is necessary. Thus, the evaluation criteria are summarized as two levels: “moderate abuse or higher” (moderate abuse, severe abuse, life-threatening), which requires a “proactive response by staff through home visits and investigations,” and “moderate abuse or lower” (suspected abuse, mild abuse), which does not require such a level of response. The risk factors for cases that were notified as moderate abuse or higher were examined.

### 2.2 Survey items used in the analysis

The Child Guidance Center notification survey included various information that pertained to the cases under examination. We utilized data that pertained to the following four points: (1) characteristics of the abused child and their respective household (including attributes of the child, type of abuse, severity of abuse, familial structure, and economic circumstances), (2) characteristics of the abuser (including attributes of the abuser and any physical or mental health issues they may have), (3) factors that contributed to the abuse (including circumstances of the child and their family that may have led to the abuse), and (4) damage sustained by the child as a result of the abuse (including both physical and psychological harm). The variables that were employed in the analysis are outlined below, along with the corresponding method of response or category.

(1) Characteristics of the abused child: Gender (*male/female*), Age at the time of case receipt (*numerical answer*), Identifier of initial abuse detection [*local government official or Child Guidance Center staff, police, educational institution (daycare center, kindergarten, school, or child-related facility), medical institution, abuser him/herself, other family member or relative, other, unknown*], Main type of abuse (*physical abuse, neglect, neglect of abuse by roommate, sexual abuse, psychological abuse, witnessing domestic violence*), Overlapping abuse (*No-Yes*), Severity of abuse (*suspected, mild abuse, moderate abuse, severe abuse, life-threatening*), Duration of abuse (*<1 month, 1–3 months, 3–6 months, 6 months-1 year, 1–3 years, >3 years*), Child's perception of abuse (*perceived as unjustly cruel, perceived as cruel but my fault, did not feel cruel, unable to confirm intention, unknown*), Family structure of abused child (*multiple answers for applicable members*), and Financial status of household (*public assistance household, partially tax-exempt household, taxable household, unknown*). Among main types of abuse, “witnessing domestic violence” is a category introduced in a survey conducted by the Ministry of Health, Labor and Welfare (5). It is considered a form of psychological abuse within a broader definition.
(2) Characteristics of the abuser: Relationship of the primary abuser (*select one applicable member*), age (*numerical response*), employment status (*regular employment, non-regular employment, domestic work, devoted to housework, unemployed, student, other, unknown*), and physical and mental disorders (*multiple responses, including psychosis, neurosis, personality disorder, intellectual disability, alcoholism, drug addiction, developmental disorder, physical illness*).
(3) Factors contributing to abuse: Circumstances of the abused child that may have contributed to the abuse (*up to five multiple responses, including unwanted birth, prematurity, multiple births, prolonged hospitalization at birth, experience of separation from parents, delay or disability in physical development, delay or intellectual disability in mental development, developmental disability, autism with intellectual disability, weakness, problematic behavior, personality bias, other*) and Family and household conditions that may have contributed to the abuse (*up to five multiple responses, including economic difficulties, unstable employment, single parent families, stepfamilies, domestic violence, physical and mental condition of the abuser, marital discord, family discord, isolation from neighbors and friends, young childbirth, childcare fatigue, aversion to childcare, other*).
(4) Damage caused by abuse to the child: Physical damage resulting from abuse (*with up to five multiple responses from such options as bruises, burns, stab wounds, broken bones, head trauma, sexual abuse, pregnancy, malnutrition, and delayed physical development*), Psychological damage resulting from abuse (*with up to five multiple responses from such options as delayed intellectual development, interpersonal problems, low self-esteem, strong aggression, emotional instability, anxiety, depression, emotionlessness, sleep disturbances, hyperactivity, eating disorders, asocial problem behaviors [such as truancy], antisocial problem behaviors [such as delinquency], sexual problem behaviors, self-injurious behavior, suicidal ideation, other*).

### 2.3 Statistical analysis

The severity of abuse cases, graded on a five-level scale, was used as the outcome variable in this study, with a binary variable of moderate abuse or more (= 1) and mild abuse or less (= 0) being employed. The criteria for determining the severity of abuse in this study are outlined in Table 1.

**Table 1:** Criterion of severity of child abuse (7).

Severity	Criterion
Life threatening	Danger to the child's life that is “possible” or “threatened.” i. Potential for life-threatening trauma due to physical violence ii. Possibility of death due to neglect.
Severe	Currently, there is no life-threatening situation, but it is likely to cause significant impacts on the child's health, growth, or development. Intervention (e.g., supervised visit, temporary separation, or hospitalization) is required to protect the child.
Moderate	Currently, there are no traumas or nutritional disorders severe enough to require hospitalization. However, in the long run, it is concerning that this may remain a serious issue in formation of the child's personality. Without intervention, no further improvement can be expected.
Mild	There is actual abuse of the child, and it has been perceived by the parents or those around them. However, there is a certain level of control, or it is considered temporary, and there is no serious pathology in the parent-child relationship. However, parents should still be consulted.
Suspected	There is no abusive behavior present, but the parents express fear of potentially abusing the child, such as statements like “I’m going to hit my child” or “I do not want to take care of my child.”

Multiple logistic regression analyses were conducted for each age group dataset, with the severity of abuse as the outcome variable and other variables as explanatory variables. The analysis employed a backward stepwise approach using the method of decreasing variables (likelihood ratio). Additionally, cumulative scores for physical and mental problems of the abuser, circumstances that led to abuse of the child, circumstances that led to abuse within the family, physical damage that was caused by the abuse, and psychological damage that was caused by the abuse were calculated and included in the analysis simultaneously, given the wide range of items.

Two-thirds of the cases were randomly selected from the dataset for model building (0–3 years old: *N* = 1,150; 4–6 years old: *N* = 968), and the remaining one-third was used for model validation (0–3 years old: *N* = 618; 4–6 years old: *N* = 492) to assess accuracy of the prediction equations. Each sample size is smaller than the total because cases of nonresponse to the variables were excluded in the analysis. Performance of the model was evaluated by assessing discrimination using the area under the receiver operating characteristic (ROC) curve (AUC). All statistical analyses were conducted using SPSS 23 software. The level of significance was set at *p *< 0.05.

## 3 Results

### 3.1 Basic statistics

Table 2 presents basic statistics for both the infant and preschool age groups. Regardless of age group, approximately 30% of cases were classified as moderate, severe, or life-threatening abuse, and the remaining 60% were classified as mild abuse or suspected abuse. The incidence of life-threatening abuse was less than 5% for both age groups, but it was relatively higher for children aged 0–3. In terms of main types of abuse, neglect, physical abuse, and witnessing domestic violence were more prevalent across both age groups. Notably, the frequency of neglect among infants (0–3 years old) was greater than 40%. Conversely, the frequency of physical abuse was relatively higher in the preschool age group (4–6 years old).

**Table 2:** Basic statistics of data^a^.

Variable	Infants (0–3 years old) *N* = 1,868	Preschool age (4–6 years old) *N* = 1,529
Sex of abused children		
Male (%)	1,015 (54.8)	809 (52.9)
Female (%)	838 (45.2)	710 (46.4)
Age of abused children (average/SD)	1.53 (1.13)	4.97 (0.81)
Main type of abuse		
Physical abuse (%)	440 (23.9)	458 (30.4)
Neglect (%)	614 (44.9)	437 (29.0)
Neglect of abuse by roommate (%)	44 (2.4)	50 (3.3)
Psychological abuse (%)	324 (17.3)	293 (19.4)
Sexual abuse (%)	6 (0.3)	20 (1.3)
Witness of domestic violence (%)	414 (22.5)	250 (16.6)
Severity of abuse		
Life threatening (%)	66 (3.8)	17 (1.2)
Severe (%)	137 (7.9)	55 (3.8)
Moderate (%)	401 (23.1)	329 (23.0)
Mild (%)	657 (37.8)	679 (47.4)
Suspected (%)	475 (27.4)	352 (24.6)

^a^ There were several cases of unanswered. The total number of items does not match the total sample size.

### 3.2 Multiple logistic regression analysis and prediction accuracy of the model

#### 3.2.1 Infancy (0–3 years old)

The final adopted model is presented in Table 3. The results indicated that a younger age of the abused child was associated with a significantly higher risk of moderate abuse or higher. With regard to the first discoverer of abuse, the risk of moderate abuse or higher significantly increased when the discoverer was a municipal employee (municipal employee or child welfare worker) or a staff member at a medical institution compared with a neighboring acquaintance. In terms of the main types of abuse, the risk of moderate abuse or higher significantly increased when main types of abuse were physical abuse, neglect, neglect by a roommate, and witnessing domestic violence compared with psychological abuse. Furthermore, the risk of being classified as moderately abusive or higher significantly increased as the cumulative score of the abuser's physical and mental problems increased, as the cumulative score of the abused child's physical damage increased, and as the cumulative score of the abused child's psychological damage increased. Additionally, the risk of being classified as moderately abusive or higher significantly increased when the abused child's psychological damage tended to be “emotionless, unresponsive, and freezing.”

**Table 3:** Multiple logistic regression of over (1)/under (0) moderate severity of child abuse (infancy, 0–3 years old, *N* = 1,150).

Explanatory variables	OR	95% CI		*p*
Age of abused child (0–3)	0.87	0.8	1.0	**0**.**030**
First person to find abuse: ref. neighborhood				**0**.**019**
Municipal or Child Guidance Center staff	1.75	1.1	2.8	**0**.**019**
Police	1.43	0.9	2.4	0.166
Nurseries, kindergartens, schools, other facilities for children	1.42	0.8	2.5	0.226
Medical institution	2.70	1.6	4.7	**0**.**000**
Abuser himself/herself	0.95	0.5	1.9	0.886
Other family members and relatives	1.29	0.8	2.1	0.325
Others	1.35	0.6	3.3	0.511
Unknown	5.35	0.6	44.3	0.120
Main type of abuse: ref. psychological abuse				**0**.**003**
Physical abuse	1.76	1.0	3.0	**0**.**037**
Neglect	2.61	1.6	4.2	**0**.**000**
Neglect of abuse by roommate	2.34	0.9	5.9	0.069
Witness of domestic violence	2.11	1.2	3.7	**0**.**008**
Abuser's mental/physical problems (cumulative score, 0–8)	1.44	1.2	1.7	**0**.**000**
Physical damage of child (cumulative score, 0–5)	2.78	2.0	3.8	**0**.**000**
Psychological damage of child (cumulative score, 0–5)	1.46	1.2	1.8	**0**.**000**
No emotional reaction, tendency to freeze (0–1)	8.14	1.6	41.2	**0**.**000**

Values in bold indicate *p < 0.05. Figures in parentheses indicate the score range.

**Figure 1:**
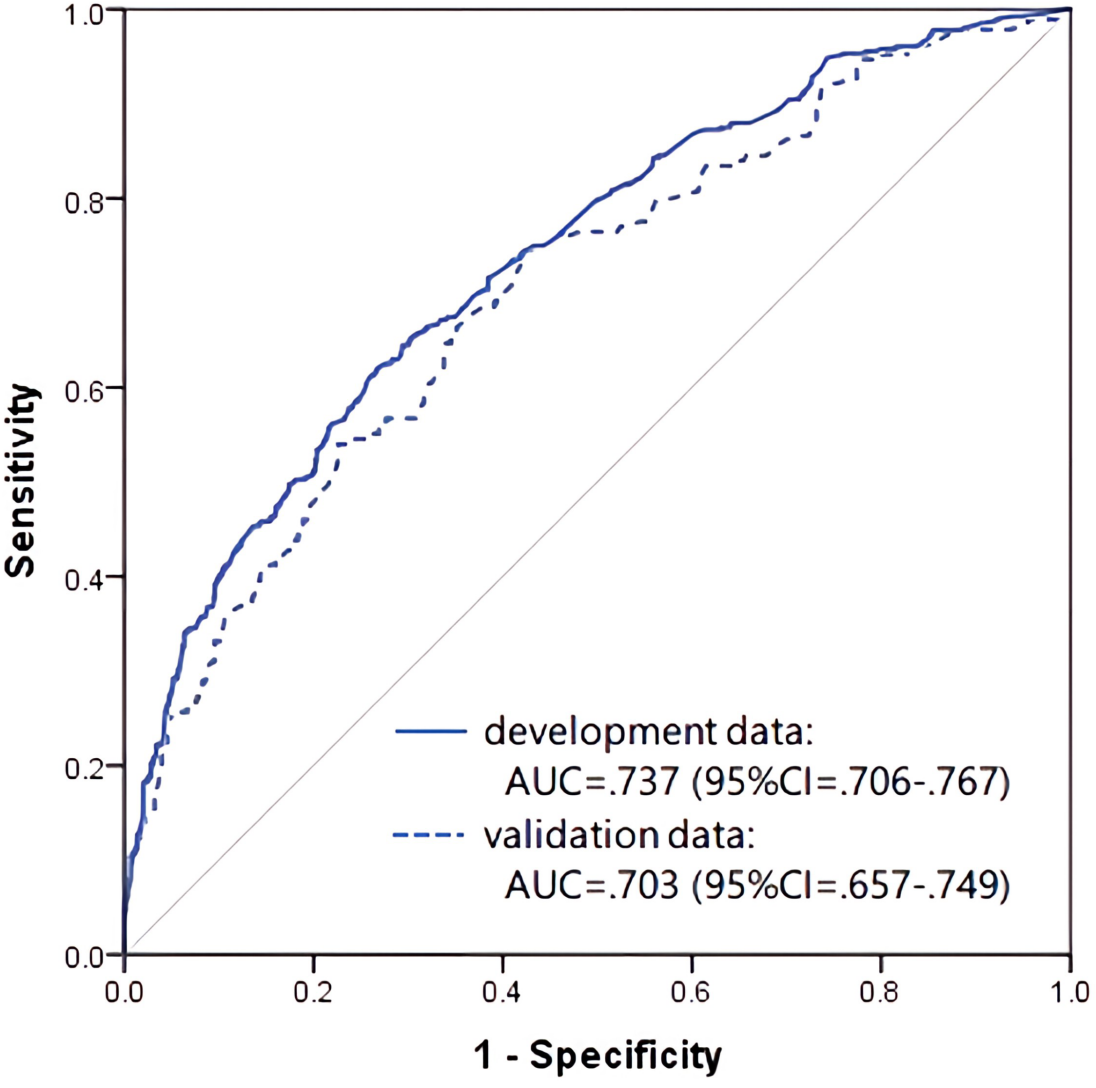
Receiver operating characteristic curve and AUC of the prediction model in infancy (0–3 years old).

The receiver operating characteristic (ROC) curve was drawn using the predicted probability values that were calculated from the adopted prediction equation, and the area under the curve (AUC) was 0.737 [95% confidence interval (CI): 0.706–0.767] in the development dataset and 0.703 (95% CI: 0.657–0.749) in the validation dataset, indicating moderate prediction accuracy (Figure 1).

#### 3.2.2 Preschool age (4–6 years old)

The final adopted model is presented in Table 4. The risk of moderate abuse or higher was significantly higher for younger children. The risk of moderate abuse or higher also significantly increased when the first discoverer of abuse was a local government employee, such as municipal staff or Child Guidance Center staff, or an educational institution staff member, as opposed to a neighboring acquaintance. Furthermore, the risk of moderate abuse or higher significantly increased when the main type of abuse was neglect and witnessing domestic violence, as opposed to psychological abuse. Additionally, the presence of overlapping abuse and the experience of separation from the abused child's guardian were both factors that significantly increased the risk of moderate abuse or higher. Furthermore, the risk of moderate abuse or higher increased as cumulative scores of the abuser's physical and mental problems and cumulative scores of the abused child's physical damage increased.

**Table 4:** Multiple logistic regression of over (1)/under (0) moderate severity of child abuse (preschool age, 4–6 years old, *N* = 968).

Explanatory variable	OR	95% CI		*p*
Age of abused child (4–6)	0.67	0.5	0.8	**0**.**000**
First person to find abuse: ref. neighborhood				**0**.**002**
Municipal or Child Guidance Center staff	3.00	1.7	5.3	**0**.**000**
Police	1.23	0.7	2.2	0.486
Nurseries, kindergartens, schools, other facilities for children	2.36	1.4	3.9	**0**.**001**
Medical institution	1.55	0.6	3.9	0.353
Abuser himself/herself	1.56	0.7	3.4	0.269
Other family members and relatives	1.19	0.7	2.0	0.526
Others	1.41	0.4	5.6	0.622
Unknown	7.19	0.8	62.6	0.074
Main type of abuse: ref. psychological abuse				**0**.**001**
Physical abuse	1.20	0.7	2.1	0.504
Neglect	2.35	1.4	3.9	**0**.**001**
Neglect of abuse by roommate	5.42	0.9	32.6	0.065
Sexual abuse	2.41	1.0	6.0	0.060
Witness of domestic violence	2.68	1.4	5.1	**0**.**002**
Multiple types of abuse	1.47	1.0	2.1	**0**.**043**
Child's experience of separation from parents	2.77	1.1	6.8	**0**.**026**
Abuser's mental/physical problems (cumulative score, 0–8)	1.94	1.6	2.4	**0**.**000**
Physical damage of child (cumulative score, 0–5)	3.31	2.3	4.8	**0**.**000**

Values in bold indicate *p < 0.05. Figures in parentheses indicate the score range.

**Figure 2:**
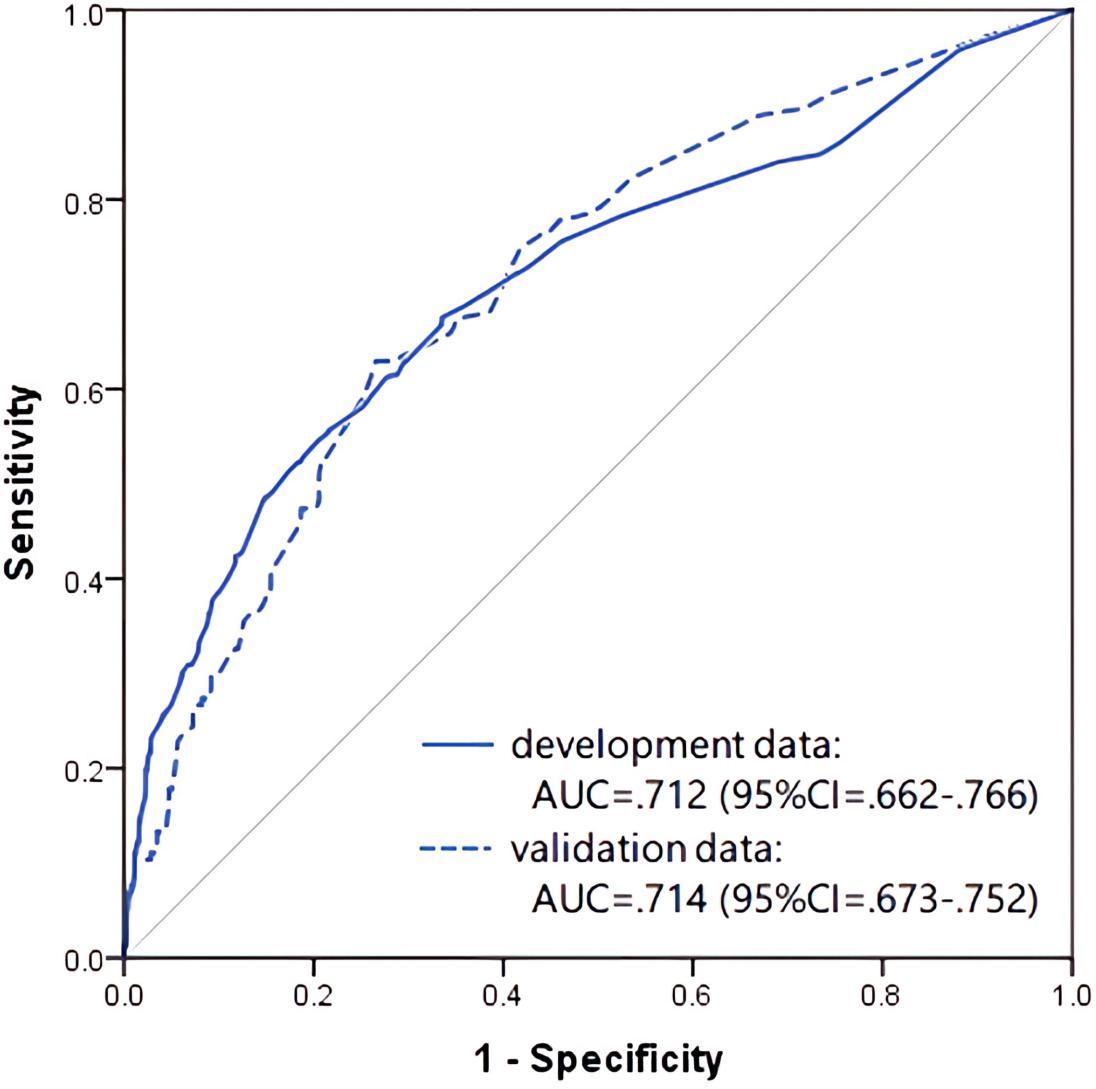
Receiver operating characteristic curve and AUC of the prediction model in preschool age (4–6 years old).

The ROC curve, generated using predicted probability values that were calculated from the adopted prediction equation, indicated moderate prediction accuracy, with an AUC of 0.712 (95% CI: 0.662–0.766) in the development dataset and 0.714 (95% CI: 0.673–0.752) in the validation dataset (Figure 2).

## 4 Discussion

The present study conducted a secondary analysis of nationwide survey data on reported cases of child maltreatment to Child Guidance Centers to identify risk factors that are closely associated with judgments of child maltreatment severity during infancy (ages 0–3) and preschool years (ages 4–6). The study also sought to develop and validate a severity risk prediction model. The model was successfully developed and validated, with risk assessment formulas that were able to predict moderate maltreatment and above for both age groups, with an accuracy of approximately 70%, indicating a certain level of validity. General characteristics of the factors that were identified as risk factors for above-moderate abuse during infancy and preschool years were the following: (1) the young age of abused children was an important risk factor, (2) as main type of abuse, severity was high, especially in cases of neglect and witnessing domestic violent, (3) the cumulative psychological or physical damage they had suffered is indicative of the greater severity of abuse, whereas the children's own problematic behavior and socioeconomic status of the family were rarely found as a risk factor, and (4) as shown in the Adverse Childhood Experiences study (24, 25), the abuser's own mental and physical problems were found as risk factors. The reason why children's own problematic behavior was not mentioned as a risk factor may be that it takes a long time for effects of abuse to manifest as problematic behavior in children (26), or children's behavioral ability may not be sufficiently developed. The reason that family socioeconomic factors are not listed as risk factors like the previous studies (27) may be that family situations function as an indirect risk factor for maltreatment severity compared with direct damage that is caused by maltreatment and thus may not have been a major risk factor.

Here, we discuss risk characteristics for each age group. In infancy (0–3 years), a younger age of the abused child was associated with a greater risk of severe abuse. This age range is a significant risk factor, especially in severe cases that lead to death. For example, in Japan in 2020, approximately 65.3% (31 cases) of the 66 annual abuse-related deaths were of children less than 1 year of age (28). This study is consistent with previous findings. The involvement of a third party, such as a medical institution, local government official, or Child Guidance Center official, in discovering and reporting the abuse may be associated with more severe cases of abuse. Medical institutions are particularly likely to have more severe cases because they are already connected with some kind of physical damage, such as trauma or malnourishment. Neglect, physical abuse, and witnessing domestic violence increased the risk of serious cases compared with psychological abuse. This may reflect the fact that physical abuse and neglect are particularly likely to be severe during infancy (29) because of physical underdevelopment and the severity of traumatic impact on memory (30). The risk of severity increased with the cumulative amount of harm suffered by the abused child, encompassing both physical and psychological dimensions. “Unfeeling, unresponsiveness, and freezing” were identified as primary indicators of psychological damage, reflecting the intensely stressful experience of abuse, including witnessing domestic violence, and the resulting emotional unresponsiveness in infants as a defensive response (31). Additionally, the abuser's own cumulative mental and physical problems were reported as contributing factors to severity, as demonstrated in the Adverse Childhood Experiences study (24, 32), which highlighted the abuser's role in exacerbating case severity through their own history of trauma and its impact on their behavior. The cumulative score of physical/mental damage to the abused child and problems of the abuser primarily influenced the risk more than individual items. These results align with another study (16) that found that cumulative risk scores were associated with temporary custody, one of the case severity factors.

For risks in preschool years (4–6 years old), the trends were overall similar to infancy. A younger age was associated with higher risk in the direction of increasing severity, a trend that was similar to infancy. The risk of abuse severity was higher when the initial notifier was local government staff, Child Guidance Center staff, or educational institution staff (daycare center, kindergarten, school, or child-related facility) compared with a neighborhood resident. This may reflect the likelihood that cases have already become serious by the time they are detected by staff of third-party specialized institutions. Neglect and witnessing domestic violence were found to increase the severity of cases compared with psychological abuse. This trend was similar to infancy. The results for physical abuse were not significant, but the cumulative score for physical damage that is attributable to abuse was a clear risk factor for severity, indicating that physical damage is associated with a higher severity of cases, regardless of the type of abuse, because of its greater impact on preschoolers. The abuser's own damage also contributed to cumulative severity, a trend that was similar to infancy. Interestingly, the past experience of separation from parents was found to be associated with increased maltreatment severity. This finding may suggest that children who experience greater severity of abuse are more likely to have been separated from their parents, perhaps due to prior instances of temporary custody (33). Compared with infancy, exposure to maltreatment may be relatively prolonged, and repeated serious problems may be more noticeable, thus posing a particular risk at this time. Abuse overlap was also found to be a risk factor for severity (34). The fact that it was reported as a risk in preschool years may reflect the fact that as children age, the range of their activities and interactions expands, making it easier to obtain detailed information about their cases.

The present study has several limitations. First, the case study on which the data were based relied on retrospective responses by Child Guidance Center staff, which may introduce bias. Second, because the data were limited to the perspective of Child Guidance Center staff, they may not capture points that might have been overlooked by them. Third, evaluations from a professional perspective (e.g., medical diagnosis) may lack validity. Fourth, there is a risk of the inconsistent application of evaluation criteria from facility to facility and respondent to respondent. Fifth, predicting risk of the severity of a case based solely on the risk items that were identified in the present study may not always be possible. Furthermore, it's important to acknowledge that our study relies on data from 2013, potentially limiting its applicability to the current child abuse situation. Given the dynamic nature of societal and legislative changes impacting child protection measures, future studies should aim to incorporate more recent data to ensure the continued relevance and accuracy of predictive models.

The present study conducted a risk assessment of child maltreatment by utilizing case data from a large-scale survey that was answered by Child Guidance Centers nationwide in Japan. We found a certain level of validity of the risk calculation formula in separate data for verification. Based on the results of the risk assessment using data from the remaining age group (7–18 years), the present findings provide valuable insights into the overall risk of abuse severity in Japan. The risk prediction equation that was derived from this study allows the prediction of case severity with moderate accuracy based on basic and peripheral information on abuse cases that are handled by Child Guidance Centers. The authors' project team developed an application to assess the severity of abuse cases by utilizing the risk assessment prediction formula to assist Child Guidance Center staff in handling abuse cases (4). Our team is working on introducing the system to local governments in Japan. However, to enhance accuracy, standardizing risk assessment criteria, incorporating longitudinal data sources, and refining the prediction model through advanced technology like machine learning are crucial. Moreover, collaboration with local government Child Guidance Centers for data updates and sharing, along with ongoing validation efforts using the latest national survey data, will further bolster the prediction model's effectiveness and contribute to advancements in child protection efforts.

## 5 Conclusion

Using a nationwide database of cases that were reported as abuse at Child Guidance Centers in Japan, we identified factors that are closely associated with child abuse severity in infancy (0–3 years old) and preschool age (4–6 years old) and constructed and validated a model to predict abuse severity. The results showed that the main risk factors for severity were young age of the child, neglect/witnessing domestic violence as the main type of abuse, the cumulative psychological and physical damage suffered by the child, and cumulative psychological and physical problems of the abuser. The validity of the prediction equation was moderate.

## Data Availability

The data analyzed in this study is subject to the following licenses/restrictions: The data that were analyzed in this study were obtained through the nationwide survey that was conducted by the National Association of Directors of Child Guidance Centers and were analyzed with permission for use. The original data are not available to the public and are difficult to use openly. For further information, please contact the Corresponding Authors (ogai.ys@md.tsukuba.ac.jp or nobumori@nifty.com).
